# Acute frataxin knockdown in induced pluripotent stem cell-derived cardiomyocytes activates a type I interferon response

**DOI:** 10.1242/dmm.049497

**Published:** 2022-10-26

**Authors:** M. Grazia Cotticelli, Shujuan Xia, Rachel Truitt, Nicolai M. Doliba, Andrea V. Rozo, John W. Tobias, Taehee Lee, Justin Chen, Jill S. Napierala, Marek Napierala, Wenli Yang, Robert B. Wilson

**Affiliations:** ^1^ Department of Pathology and Laboratory Medicine, Children's Hospital of Philadelphia, Philadelphia, PA 19104, USA; ^2^ Department of Medicine, Division of Translational Medicine and Human Genetics, University of Pennsylvania, Philadelphia, PA 19104, USA; ^3^ Institute of Diabetes, Obesity, and Metabolism, University of Pennsylvania, Philadelphia, PA 19104, USA; ^4^ Department of Genetics, Penn Genomics Analysis Core, University of Pennsylvania, Philadelphia, PA 19104, USA; ^5^ Department of Neurology, UT Southwestern Medical Center, Dallas, TX 75390, USA; ^6^ Department of Pathology and Laboratory Medicine, University of Pennsylvania, Philadelphia, PA 19104, USA

**Keywords:** Friedreich ataxia, Cardiomyopathy, mtDNA, Interferon, Innate immunity

## Abstract

Friedreich ataxia, the most common hereditary ataxia, is a neuro- and cardio-degenerative disorder caused, in most cases, by decreased expression of the mitochondrial protein frataxin. Cardiomyopathy is the leading cause of premature death. Frataxin functions in the biogenesis of iron-sulfur clusters, which are prosthetic groups that are found in proteins involved in many biological processes. To study the changes associated with decreased frataxin in human cardiomyocytes, we developed a novel isogenic model by acutely knocking down frataxin, post-differentiation, in cardiomyocytes derived from induced pluripotent stem cells (iPSCs). Transcriptome analysis of four biological replicates identified severe mitochondrial dysfunction and a type I interferon response as the pathways most affected by frataxin knockdown. We confirmed that, in iPSC-derived cardiomyocytes, loss of frataxin leads to mitochondrial dysfunction. The type I interferon response was activated in multiple cell types following acute frataxin knockdown and was caused, at least in part, by release of mitochondrial DNA into the cytosol, activating the cGAS-STING sensor pathway.

## Teaser

**Summary:** In induced pluripotent stem cell-derived cardiomyocytes, acute frataxin knockdown, which compromises iron-sulfur cluster biogenesis, is associated with mitochondrial dysfunction and activation of a type I interferon response by cytosolic mitochondrial DNA.

## INTRODUCTION

Friedreich ataxia (FRDA) is an autosomal-recessive, inherited neuro- and cardio-degenerative disorder. The neurological symptoms – ataxia, areflexia and sensory loss – usually present in the first decade of life and are relentlessly progressive (Dürr et al., 1996). FRDA is caused by decreased expression of frataxin, a nuclear-encoded, mitochondrial protein (Pandolfo, 2008). Frataxin functions primarily in the biogenesis of iron-sulfur clusters (Gervason et al., 2019), prosthetic groups that are found in proteins involved in many biological processes (Rouault, 2019). Decreased expression of frataxin in FRDA is due to triplet (GAA) repeat expansions in the first introns of both alleles of the *FXN* gene (Campuzano et al., 1996), leading to epigenetic changes that decrease transcription (Gottesfeld, 2007). In a small percentage of cases, there is a repeat expansion on one allele and a point mutation on the other allele; point mutations result in proteins that are unstable or poorly functional (Galea et al., 2016). The age of onset and the severity of the disease correlate with the length of the shorter repeat expansion (Dürr et al., 1996). A large majority of patients develop extra-neurological symptoms such as scoliosis, diabetes and cardiomyopathy, which is the leading cause of premature death (Lynch et al., 2012). FRDA-associated cardiomyopathy has been characterized through electrocardiographic and magnetic resonance imaging (MRI) studies as a progressive, concentric hypertrophy of the left ventricle, with increased thickness of the ventricular walls and a mostly conserved ejection fraction, which can progress, in some cases, to a dilated cardiomyopathy (Weidemann et al., 2003). Arrhythmias may also contribute to heart failure (Weidemann et al., 2003). Post-mortem analyses have shown loss of contractile sarcomeres, diffuse fibrosis and regions of necrosis in the heart, as well as significant inflammation (Koeppen et al., 2015).

Murine models of FRDA recapitulate only partially the specific features of FRDA-associated cardiomyopathy. Hypertrophic cardiomyopathy in a mouse model in which frataxin was conditionally knocked out in cardiomyocytes resulted in a sudden onset and rapid death (Puccio et al., 2001). Evidence of cardiac pathology has been found in a doxycycline-induced knockdown model (Chandran et al., 2017); however, doxycycline is itself a mitochondrial toxin and could contribute to phenotypes in synergy with decreased frataxin. In other mouse models – the YG8R mouse, which expresses a transgenic human frataxin containing a (GAA) repeat expansion in a background null for murine frataxin (Al-Mahdawi et al., 2006), and repeat-expansion knock-in models [knock-in, knockout (KIKO)/knock-in, knock-in (KIKI)] (Miranda et al., 2002) – little or no cardiomyopathy is evident.

The mechanisms underlying the pathophysiology of FRDA cardiomyopathy are still poorly understood and have been studied mostly in rodent-cell models (Abeti et al., 2018; Obis et al., 2014; Purroy et al., 2018). The successful reprogramming of patient-derived fibroblasts into induced pluripotent stem cells (iPSCs) (Ku et al., 2010; Liu et al., 2011) and the successful differentiation of these iPSCs into induced cardiomyocytes (iCMs) (Hick et al., 2013), opened up the possibility of extending the studies of cardiomyocytes in murine FRDA models to human FRDA cardiomyocytes. However, the number of lines generated to date is still too small to allow a rigorous study comparing FRDA iCMs to normal controls.

To decrease the many variables in the study of FRDA iCMs (e.g. GAA repeat lengths, age, cardiomyopathy stage), Li et al. created isogenic pairs using genome editing to shorten GAA repeat expansions (Li et al., 2015). A comparison of one control line, one FRDA line and one isogenic ‘corrected’ line showed transcriptomic changes associated with hypertrophic cardiomyopathy in the FRDA cardiomyocytes (Li et al., 2019). However, very few isogenic pairs are available, and even the most efficient differentiation protocols result in different degrees of differentiation.

To overcome some of these limitations, we developed a novel model to study FRDA cardiomyopathy: we generated isogenic pairs by differentiating wild-type iPSCs into cardiomyocytes and knocking down frataxin post-differentiation. This approach controls not only for genetic background, but also for small differences in differentiation. Transcriptome analysis using four biological replicates identified pathways that are highly affected by frataxin knockdown. We confirmed our transcriptome analysis by quantifying mitochondrial electron transport chain (ETC) dysfunction in cells with low frataxin. We also found that frataxin knockdown contributes to the activation of the type I interferon pathway. We confirmed the interferon activation in multiple FRDA cell models and found that this activation is at least partially due to an increase in cytosolic mitochondrial DNA (mtDNA), which is detected by the cGAS-STING sensor pathway.

## RESULTS

### A novel isogenic model to study FRDA cardiomyopathy using iPSC-derived cardiomyocytes

Four apparently healthy and previously characterized human iPSC lines (SV20, GM00942, GM21808 and GM08399) (Li et al., 2015; Shanmughapriya et al., 2018; Yang et al., 2008) were successfully differentiated into cardiomyocytes (iCMs): cells were beating by day 12 post-differentiation and were routinely ≥90% positive for cardiac troponin by day 25 post-differentiation (Fig. S1A,B). To generate an isogenic model system, the cells were seeded in their final vessel and transfected with a siRNA targeting frataxin mRNA (siFxn) or with a control siRNA (sc), following the scheme shown in Fig. S1C. Forty-eight hours after the first transfection, frataxin protein levels were ∼50% of control levels (Fig. S1D). Cells were transfected again on day 5. Forty-eight hours after the second transfection (day 7), frataxin protein levels were 20-35% of control levels (Fig. S1D). No cell death was observed after the first transfection; at day 7, however, there was ∼20-30% death in the cells transfected twice with siFxn. We extracted RNA from four independent lines for transcriptome analysis and focused our analysis on samples obtained at day 7; principal component analysis showed a clear separation of control cells from cells in which frataxin was knocked down (Fig. S1E).

### Transcriptome analysis and mitochondrial dysfunction

**Fig. 1.**
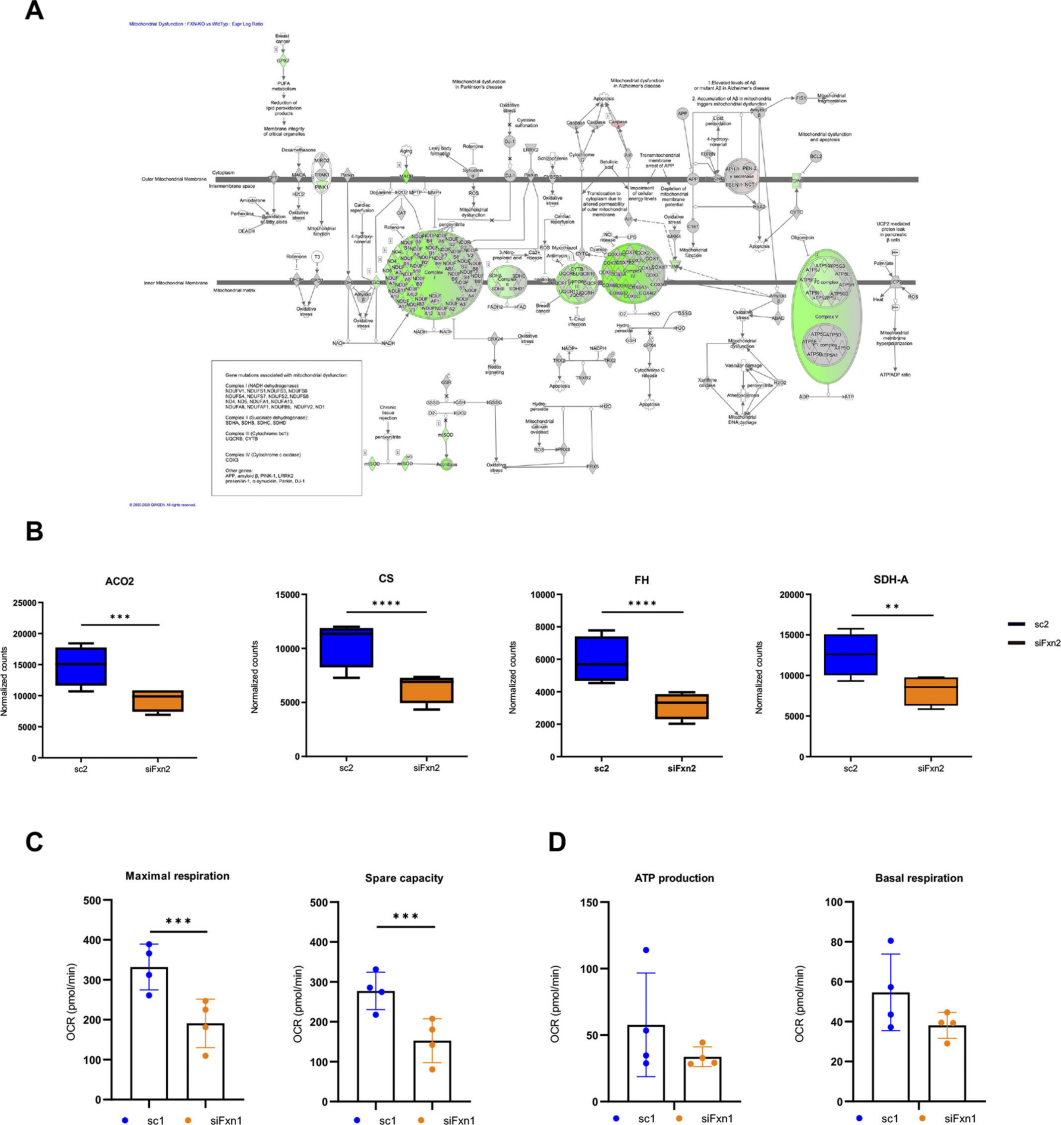
**Frataxin knockdown impairs mitochondrial respiration in induced cardiomyocytes (iCMs).** (A) Ingenuity Pathway Analysis (IPA) representation of electron transport chain (ETC) subunits downregulated following frataxin knockdown in iCMs (shown in green). Number of biological replicates=4. (B) Box and whisker plots showing normalized counts for aconitase (ACO2), citrate synthase (CS), fumarate hydratase (FH) and succinyl dehydrogenase (SDHA) in iCMs transfected twice with control sc2 (blue) or siFx2 (orange). Boxes represent the 25-75th percentiles, and the medians are indicated. The whiskers show the furthest data point within 1.5 times the interquartile range from each box end. Number of biological replicates=4; ****P-adj<0.0001; ***P-adj<0.001; **P-adj<0.01 using DESeq2 (Love et al., 2014). (C) Average maximal respiratory capacity (left) and spare capacity (right) are lower in iCMs transfected once with siFxn (orange) versus control (sc1; blue). Data are represented as weighted averages±composite s.d. calculated for the four biological replicates. ***P<0.001 (unpaired, two-tailed Student's *t*-test). (D) Average ATP production (left) and basal respiration (right) are statistically unchanged in iCMs transfected once with siFxn (orange) versus control (sc1; blue). Data are represented as weighted averages±composite s.d. calculated for the four biological replicates.

Ingenuity Pathway Analysis (IPA; see Materials and Methods) of the transcriptome data identified ‘Mitochondrial Dysfunction’, ‘TCA cycle II (Eukaryotic)’ and ‘Oxidative Phosphorylation’ among the canonical pathways most affected by frataxin knockdown [adjusted *P*-values (*P*-adj)<0.0001]. There was a downregulation of mRNAs encoding multiple subunits of each complex of the electron transport chain (ETC; represented graphically in Fig. 1A) as well as those encoding several enzymes involved in the tricarboxylic acid (TCA) cycle (*ACO2*, *CS*, *FH*, *IDH3A*, *DHTKD1*, *SDHA*, *OGDH* and *OGDHL*). Fig. 1B shows the normalized counts for mitochondrial aconitase (*ACO2*), citrate synthase (*CS*), fumarate hydratase (*FH*) and succinate dehydrogenase (*SDHA*). To confirm compromised mitochondrial function, we used the Seahorse assay to quantify oxygen consumption rate (OCR) at the basal level and after inhibition of specific ETC complexes in cells in which frataxin was knocked down. We initially choose to study the samples at day 7, in cells transfected twice with siFxn; however, as Seahorse data were collected on cell aggregates, we found that the cell death that occurs in the course of the longer experiment, when frataxin levels are at their lowest, made the measurements unreliable. As the transcriptomic data predict a quite severe mitochondrial impairment at day 7, we tested whether a change in mitochondrial function could also be detected when frataxin levels were acutely knocked down to only 50% of control. Representative results from one cell line are shown in Fig. S2A; after a single transfection with siFxn, basal respiration, maximal respiration and ATP production in the iCMs were all significantly decreased (Fig. S2B). In the four biological replicates, maximal respiration was 30%, 58%, 69% and 79% of that of controls for the lines SV20, GM00942, GM21808 and GM08399, respectively (Fig. S2C, top), and spare respiratory capacity was 28%, 54%, 65% and 75% of that of controls for the same four lines, respectively (Fig. S2C, bottom). The average decrease in maximal respiration for the four biological replicates was 41% (*P*<0.001, Fig. 1C, left), and the average decrease in spare respiratory capacity was 44% (*P*<0.001, Fig. 1C, right) of that of controls. ATP production decreased to 28%, 83% and 81% of control values for lines SV20, GM00942 and GM21808, respectively, but did not change in line GM08399; the average for the four biological replicates was a 26% decrease, which did not reach statistical significance (*P*=0.298, Fig. 1D, left). Changes in basal respiration, even when statistically significant, were small and likely reflected slight differences in cell density (Fig. 1D, right). Extracellular acidification rates were substantially unchanged in all lines.

### Mitochondrial morphology and copy number

Staining iCMs with the mitochondrial dye Mitogreen showed that the mitochondria were mostly perinuclear, as expected (Fig. S3A). RNA-sequencing data did not show any significant difference in the expression of *PGC1alpha* (also known as *PPARGC1A*), a master regulator of genes involved in mitochondrial biogenesis, or in the expression of PGC1alpha targets transcription factor A, mitochondrial (*TFAM*) and nuclear respiratory factor 1 (*NRF1*) (reviewed in Scarpulla, 2011). Transcription factors involved in the regulation of autophagy – *TFEB*/*TFE3*, *FOXO1/3/4/6* and *BNIP3* (Di Malta et al., 2019) – were also unchanged. Three autophagy markers – *ATG3*, p62/*SQSTM1* and *FUNDC1* – were upregulated in the hearts of conditional *FXN* knockout mice (Huang et al., 2013); in our system, *ATG3* and p62/*SQSTM1* expression were unchanged and *FUNDC1* was downregulated (log_2_-fold change=−0.65039, 30% of control, *P*-adj=0.0007). In *Caenorhabditis elegans*, downregulation of frataxin resulted in increased lifespan and increased autophagy mediated by *pdr-1*, *pink-1* and *dct-1*, the *C. elegans* homologs of *PRKN*, *PINK1* and *BNIP3*, respectively (Schiavi et al., 2015). In our system, we did not see any changes in transcriptional levels of *PRKN* and *BNIP3*, whereas *PINK1* was significantly downregulated (log_2_-fold change=−0.64046, 64% of control, *P*-adj=2.09×10^−5^).

Changes in mitochondrial copy number expressed as a ratio of mtDNA to nuclear DNA (nDNA) have been linked to cardiovascular disease (Castellani et al., 2020b). In the hearts of FRDA patients (Bradley et al., 2000) and KIKO mice (Jasoliya et al., 2017), this ratio was decreased. Frataxin knockdown induced a steep decrease of this ratio in human fibroblasts (Jasoliya et al., 2017). We measured the mtDNA/nDNA ratio using three different mitochondrially encoded genes. The mtDNA/nDNA ratio in iCMs in which frataxin was knocked down decreased compared to that in control cells in all four lines (Fig. S3B; *P*<0.0001 by two-way ANOVA).

### Mitochondrial membrane potential

To measure whether frataxin knockdown affected mitochondrial membrane potential (MMP), we used the fluorescent dye JC-1. The import of JC-1 into mitochondria is dependent on the MMP and results in a shift in the ratio of mitochondrial (red) fluorescence to cytosolic (green) fluorescence, thereby providing an indirect measure of mitochondrial depolarization. A representative JC-1 stain is shown in Fig. S3C. The differences in corrected total cell fluorescence (CTCF) ratios, induced by frataxin knockdown, showed a consistent trend toward hyperpolarization, ranging from +2% (SV20) to +15% (GM21808) and +16% (GM00942), but did not reach statistical significance (with the exception of line GM08399 at +22%, *P*=0.02). The average of the four biological replicates showed no significant difference between frataxin knockdown cells and controls (*P*=0.1392, Fig. S3D).

### Type I interferon activation

IPA analysis identified activation of the type I interferon pathway as the pathway most affected by frataxin knockdown in iCMs. IFNβ triggers the transcription of multiple interferon-stimulated genes (ISGs) through binding to the INFβ receptor and the engagement of STATs. INFβ was identified as an upstream regulator by IPA analysis (*z*-score=6.59) and was increased ∼170-fold in iCMs following frataxin knockdown (log_2_-fold change=7.41). Upregulation of INFα/β is canonically associated with bacterial or viral infections, during which foreign DNA or RNA is recognized by specific pattern-recognition receptors, leading to upregulation of *IFNB1* transcription (Brubaker et al., 2015).

**Fig. 2.**
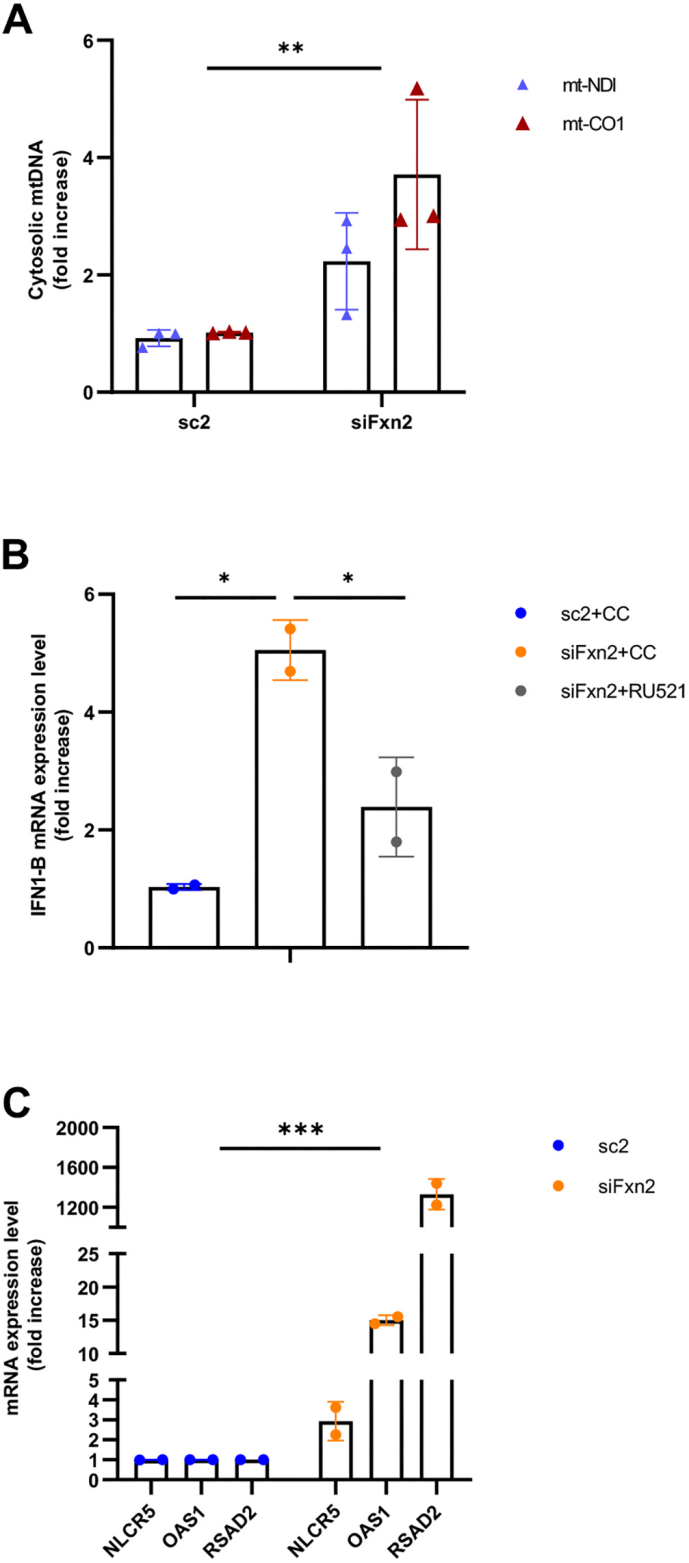
**Type I interferon response associated with frataxin knockdown.** (A) Cytosolic mitochondrial DNA (mtDNA) measurements. Cytosolic mtDNA was extracted from NBT cells transfected twice with control (sc2, left) or siFxn (siFxn2, right). *MTND1* (blue) and *MTCO1* (red) were amplified, and their concentrations were normalized to 18S DNA. Differences between the sc2 and siFxn2 measurements are statistically significant (**P=0.0019 by two-way ANOVA). (B) *INF1B* expression levels increase in NBT cells transfected twice with siFxn (siFxn2, orange) or control (sc2, blue). Treatment with the cGAS inhibitor RU521 at 2 µM after each transfection decreased *IFN1B* expression. CC indicates drug vehicle, 0.1% dimethyl sulfoxide (DMSO). Differences among columns are statistically significant: *P<0.05 by one-way ANOVA with Bonferroni post-test correction. (C) Upregulation of ISG genes *NLRC5*, *OAS1* and *RSAD2* in NBT cells transfected with siFxn (siFxn2, orange) or control (sc2, blue). Differences between sc2 and siFxn2 are significant: ***P<0.001 by two-way ANOVA. Data show the mean±s.d. of three (A) or two (B,C) independent experiments, each one at least in duplicate.

Recent studies suggest that interferon responses can be activated by cell-intrinsic events such as senescence (De Cecco et al., 2019). In particular, release of mtDNA into the cytosol has been shown to potentiate type I interferon responses through activation of the cGAS-STING recognition system (Sun et al., 2013). To test the hypothesis that frataxin knockdown, and the associated mitochondrial dysfunction we observed, lead to interferon activation through the release of mtDNA into the cytosol, we used cell lines that scale sufficiently, which simultaneously allowed us to determine whether the phenotype generalizes to other cell types. We started with the human myoblast cell line, NBT. Knocking down frataxin in NBT cells, as we did in the iCMs (Fig. S1C), replicated the results we obtained using iCMs: compromised ETC functionality as assessed by Seahorse analysis, unaffected mitochondrial morphology, downregulation of mitochondrial aconitase and decreased mitochondrial copy number (Fig. S4). In NBT cells, frataxin knockdown resulted in a significant increase in cytosolic mtDNA (Fig. 2A). *IFNB1* expression increased 5-fold (Fig. 2B). Treatment after each transfection with 2 µM RU251, an inhibitor of the cytosolic DNA sensor cGAS, decreased the expression of *INF1B* by 50% compared to cells treated with carrier control. Frataxin knockdown also led to the upregulation of three ISGs: *NLRC5*, *OAS1* and *RSAD2* (3-, 15- and 1300-fold, respectively; Fig. 2C), in agreement with the iCM transcriptomic data. We repeated these experiments in human, apparently healthy, primary fibroblasts and got the same results: increased cytosolic mtDNA, *INF1B* upregulation sensitive to RU251 treatment and upregulation of ISGs (Fig. S5).

**Fig. 3.**
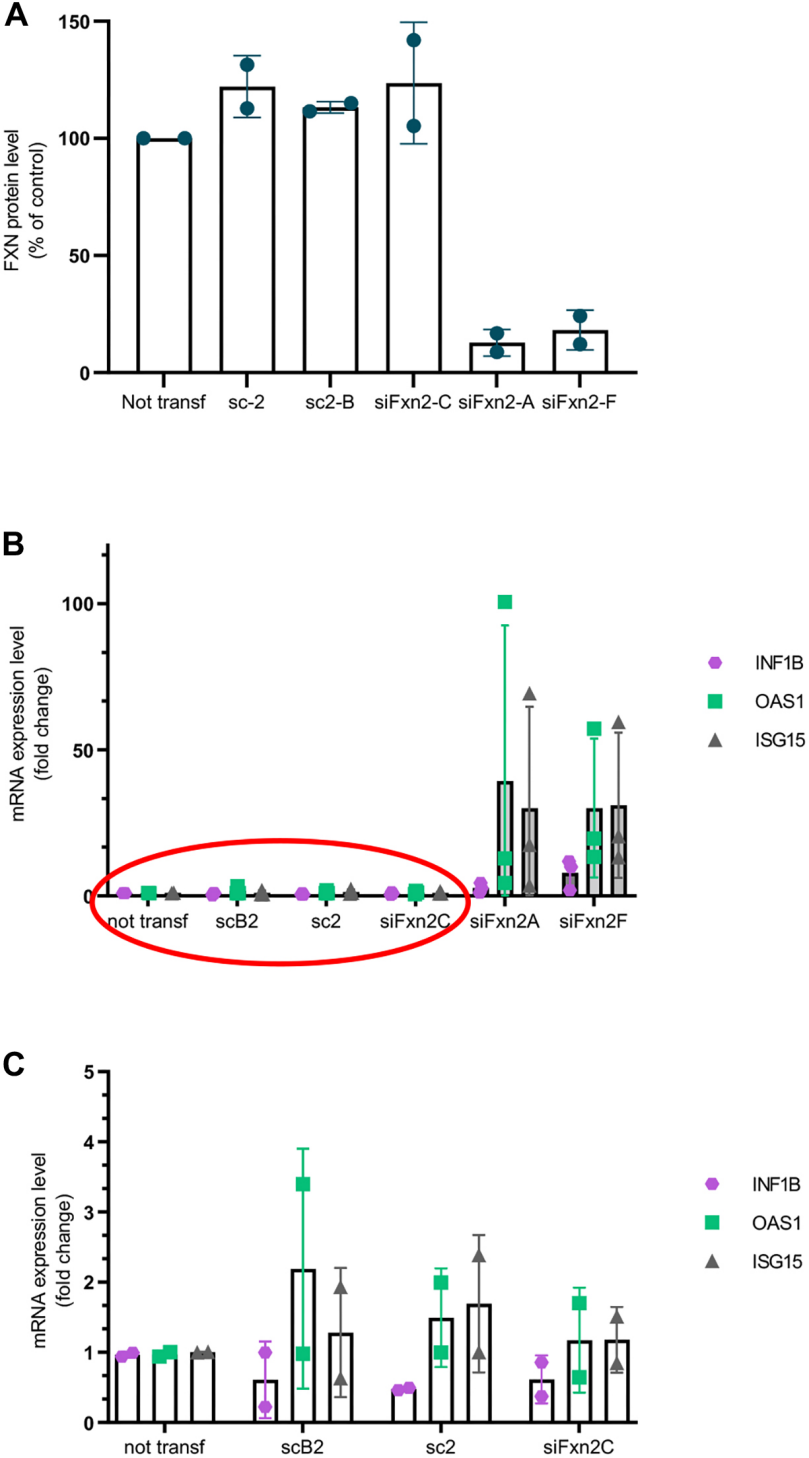
**Type I interferon response is lower in transfection controls.** (A) *FXN* expression levels in NBT cells not transfected, transfected twice with control siRNAs (scB2 and sc2), transfected with a targeted siRNA that failed to knock down frataxin (siFxn2C), or transfected with targeted siRNAs that succeeded in knocking down frataxin (siFxn2A, siFxn2F). (B) *INF1B*, *OAS1* and *ISG15* expression levels are higher in cells in which *FXN* was knocked down successfully*.* (C) Blow-up of the oval from B. Data are represented as mean±s.d. from two independent experiments (A-C).

Although we used control siRNAs in all of these experiments, we considered the possibility that the transfected siRNAs we used to knock down frataxin might trigger an interferon response (though oligonucleotides with fewer than 30 bases should be less able to do so (Morral and Witting, 2012). To confirm that the *IFN1B* upregulation we observed with frataxin knockdown is siRNA-sequence independent, we tested four more constructs targeting frataxin mRNA and added another commercially available control. One of the siRNAs targeting frataxin (siFXN2C) failed to knock down frataxin expression appreciably, whereas the others knocked down frataxin expression >80% (Fig. S6, top). Upregulation of *IFN1B*, and the ISGs *OAS1* and *ISG15* (Fig. S6, middle) was seen only in the cells transfected with the four constructs that downregulated frataxin expression level below 20% of that of control (Fig. 3B). These differences were statistically significant (Fig. S6, bottom).

To further address the possible contribution of transfected siRNAs to *IFN1B* activation in our system, we compared cells transfected with our control siRNAs to untransfected cells. Fig. 3A shows frataxin protein level in the cells not transfected, as well as in cells transfected with the constructs described above, and the corresponding upregulation of *INFB1* as well as ISGs such as *OAS1* and *ISG15* (Fig. 3B). We found that *OAS1* and *ISG15* were upregulated up to 3-fold in cells transfected with control siRNAs; however, *IFN1B* expression was decreased 55-80% in transfected cells versus untransfected cells (Fig. 3C). Similar levels of ISG upregulation and *INFB1* downregulation were seen in cells transfected with the frataxin-targeted siRNA that failed to knock down frataxin (Fig. 3C). Although the upregulation of these genes did not reach statistical significance, there was a trend in each experiment for both genes (Fig. 3C). Regardless, even if siRNA transfection itself contributes slightly to upregulation of ISGs, this activation is an order of magnitude lower than that seen with frataxin knockdown. siRNAs activate retinoic acid-inducible gene I (RIG-I)-like receptors (RLRs), which upregulate *IFNL1* (Brubaker et al., 2015), a type III interferon. IFNL1 is expressed in iCMs (log_2_-fold change=6.76; *P*-adj=2.22×10^−5^), NBT cells and fibroblasts following frataxin knockdown, but is not detected in cells transfected with sc2 control.

Cytosolic double-stranded mtRNA can also contribute to an innate immune response. Release of double-stranded mtRNA into the cytosol from dysfunctional mitochondria is dependent on the RNA helicase SUV and the polynucleotide phosphorylase PNPase, encoded by the *SUPV3L1* and *PNPT1* genes, respectively (Dhir et al., 2018). In our iCM transcriptomic data following frataxin knockdown, *SUPV3L1* mRNA levels were unchanged whereas *PNPT1* mRNA levels were upregulated (fold change=4.93, *P*-adj=1.64×10^−15^, Fig. S7A). Knocking down frataxin in NBT cells and in primary human fibroblasts also resulted in upregulation of *PNPT1* mRNA (Fig. S7B). Using an antibody specific to double-stranded RNA (dsRNA), we did not detect a significant increase in dsRNA in NBT cells in which frataxin was knocked down versus cells transfected with a control siRNA, and we did not pursue this further. These data suggest that mitochondrial dsRNA is not a significant contributor to phenotypes in our models.

**Fig. 4.**
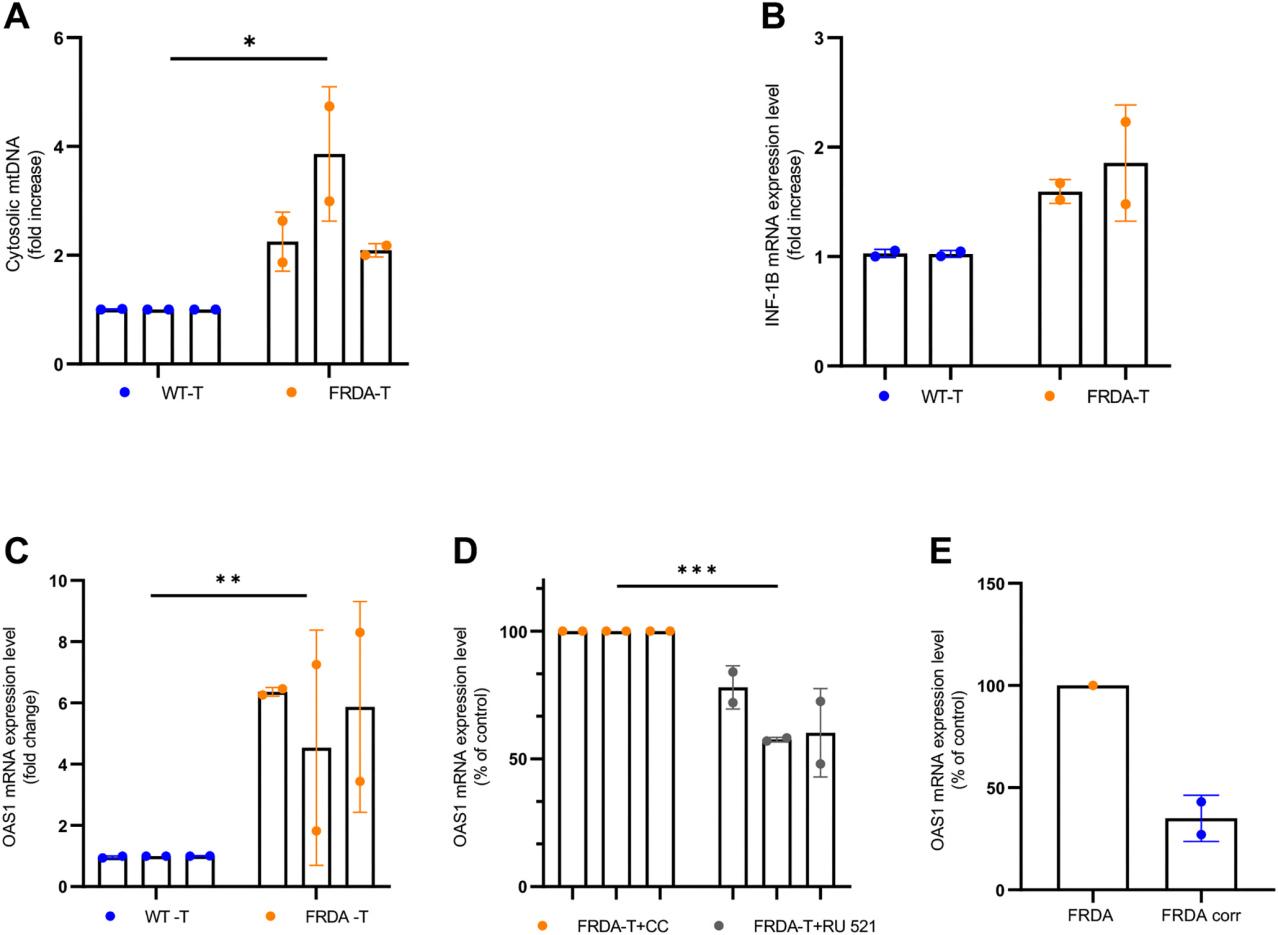
**Concentration of cytosolic mtDNA is higher in frataxin-deficient cells.** (A) Cytosolic mtDNA was extracted from three immortalized healthy controls (3348-T, 3956-T and 8399-T) and three immortalized FRDA patient-derived fibroblasts (4675-T, 6247-T and 3665-T). *MTND1* was amplified and normalized using 18S DNA. Differences between the averages of biological replicates for wild-type (WT-T) and FRDA cells were statistically significant: *P<0.05 by two-way ANOVA. (B) *INF1B* expression trends higher in immortalized FRDA patient derived fibroblasts versus apparently healthy (WT-T) counterparts. *INF1B* expression was not detectable in control line 8399-T. (C) *OAS1* expression is higher in immortalized FRDA patient-derived fibroblasts (4675-T, 6247-T and 3665-T) versus control fibroblasts (3348-T, 3956-T and 8399-T). Differences between the averages of biological replicates for WT and FRDA cells were statistically significant: **P=0.009 by two-way ANOVA. (D) *OAS1* expression is sensitive to 2 µM RU521 treatment in immortalized FRDA patient-derived fibroblasts (4675-T, 6247-T and 3665-T). CC indicates vehicle control, 0.1% DMSO. ***P=0.0003 by two-way ANOVA. (E) *OAS1* expression is higher in iCMs derived from a FRDA patient than in iCMs derived from the same patient's cells ‘corrected’ to be heterozygous. Two independent clones of the corrected lines were used. Data represent the mean±s.d. of two independent experiments, each one at least in duplicate.

Having confirmed that the activation of an interferon response follows acute frataxin knockdown in fibroblasts, we then quantified cytosolic mtDNA in primary human fibroblasts immortalized by stably integrating the gene encoding human telomerase (which we indicate by adding ‘-T’ to the name). These fibroblasts were derived from apparently healthy cells (3348-T, 3956-T and 8399-T) or from FRDA patients (4675-T, 6247-T and 3665-T). Cytosolic mtDNA (specifically the *MTND1* gene) concentrations were, on average, 2.76-fold higher in the FRDA cells (Fig. 4A). *IFN1B* expression trended higher in the FRDA cells (Fig. 4B); however, in one control line the expression levels were barely detectable, which did not allow for reliable quantification. *OAS1* expression levels were 5.5-fold higher in FRDA cells (Fig. 4C). Treatment with RU521 at 2 µM for 24 h significantly decreased *OAS1* expression to 65% of control values (Fig. 4D). Finally, we quantified the expression level of *OAS1* in one isogenic pair of iCMs (Li et al., 2019). We found that, in the two clones in which one of the two GAA-repeat expansions had been removed, *OAS1* expression was decreased 34%, on average, compared to the isogenic, FRDA patient-derived line (Fig. 4E). Taken together, these data suggest that release of mtDNA into the cytosol due to low frataxin can contribute to an innate immune response.

## DISCUSSION

To elucidate the molecular mechanisms underlying FRDA-associated cardiomyopathy, we developed a novel isogenic model to study FRDA cardiomyopathy, knocking down frataxin in iCMs post-differentiation. Paired, isogenic cell lines for the study of FRDA have been generated by genome editing GAA repeat expansions in patient-derived cells (Li et al., 2019, 2015; Mazzara et al., 2020); in these model systems, the FRDA cells were genome edited prior to iPSC induction, a multi-step process that includes the picking of individual clones and months of culture. The primary advantage of our model is that it eliminates the variance associated with the independent processing of the pair: when we knock down frataxin post-differentiation, the iCMs are not only isogenic, but also identical in terms of passage number and degree of differentiation, with the same percentage of cardiac troponin-positive cells, etc., variables that are not controlled in genetically modified isogenic pairs. Other advantages of our system are that (1) the yield of iCMs from wild-type iPSCs is generally higher, facilitating the study of low-abundance proteins, and (2) more biological replicates are available, improving the discernment of differences. The major limitation of our system is that the decrease in frataxin is acute, which may preclude the establishment of some compensatory changes normally present *in vivo*; this might, however, reveal underlying biochemical abnormalities that would otherwise be masked by the compensatory changes.

We analyzed transcriptomic data from four pairs of isogenic iCMs in our model system using IPA. Unsurprisingly, among the pathways most affected by frataxin knockdown was mitochondrial dysfunction, which is a hallmark of FRDA evident in all models of the disease. Decreased enzymatic activity of aconitase and complexes of the ETC have been reported from cardiac biopsies of patients (Rötig et al., 1997), as well as in MCK conditional heart knockout mice (Puccio et al., 2001); mitochondrial ATP production was decreased in ^31^P-MRI analyses of the skeletal muscle (Lodi et al., 1999) and hearts (Lodi et al., 2003) of FRDA patients, and the activity of aconitase was decreased in the hearts of a murine frataxin knockdown model (Chandran et al., 2017).

To evaluate the effect on mitochondrial function of the significant downregulation we observed transcriptomically, we used Seahorse analysis of OCRs. Initially, we focused our study on the time point at which frataxin levels were lowest, but we found that the cell death associated with these levels precluded a reliable analysis in iCMs. We then tested whether there was a phenotype at an earlier time, when frataxin was knocked down 50% (on average) and there was no cell death. We found a significant decrease in the ability of mitochondria in these cells to respond to energy demand – maximal and spare respiratory capacity were decreased even in the one line (line 8399) in which ATP production was unchanged. Cells with low frataxin did not ramp up their glycolysis, and coupling efficiencies were unchanged in all but one line, which had the most severe decrease in all parameters and in which coupling efficiency decreased ∼50%. A 50% decrease in frataxin is found in FRDA carriers who are asymptomatic, so it may be surprising that a 50% decrease causes a phenotype in iCMs. However, the decrease in frataxin in the iCMs was achieved in 48 h, which might be too short a time to develop compensatory mechanisms that may play a role *in vivo*. In addition, although the Seahorse analysis revealed a compromise in functionality, the cells survived the stress of the experiment and ATP production was statistically unchanged (taking into account the results with all four lines). A less likely alternative is that the phenotype we see is driven by a subpopulation of cells in which frataxin levels are well below 50% but still high enough to maintain cell viability. For each cell line, the results were highly robust. We did see variance between the different lines, most likely due to differences in genetic background, sex and/or age of the cells we used. The results of our OCR analyses were similar to those reported for FRDA iPSC-derived sensory neurons (Igoillo-Esteve et al., 2020) and were sufficiently robust and reproducible for testing experimental therapeutics.

Unexpectedly, despite decreased maximal respiratory capacity, MMP was unchanged. Decreased frataxin expression has been associated with a lower MMP in several FRDA neuronal models (Bolinches-Amoros et al., 2014; Mincheva-Tasheva et al., 2014; Mollá et al., 2017). However, the MMP was unchanged after frataxin knockdown in neonatal rat cardiomyocytes (Obis et al., 2014), suggesting the possibility of different mechanisms in different cell types. It is also possible that the model systems and techniques in these studies are so different that the results cannot be easily compared. Although the MMP in our system was statistically unchanged, we did observe a trend toward hyperpolarization. Hyperpolarization has been proposed to prevent the formation of the permeability transition pore, which may keep cells alive or precede apoptosis (Iijima et al., 2003).

Concomitant with decreased functionality of the ETC, we also observed a transcriptional downregulation of multiple subunits of every complex of the ETC, as well as of TCA cycle enzymes, and proteins that do not contain iron-sulfur clusters, such as *VDAC2* and *SOD2*. A broad downregulation of transcription seems to be a common result of low frataxin, having been noted in multiple tissues of KIKO mice (Coppola et al., 2009), in primary human FRDA fibroblasts (Napierala et al., 2017) and in FRDA peripheral blood (Nachun et al., 2018). In another study, multiple subunits of complex I, both nuclear and mitochondrially encoded, were downregulated in FRDA peripheral blood (Salehi et al., 2014). It is possible that transcriptional downregulation is a homeostatic response to decreased iron-sulfur cluster biogenesis. Alternatively, or in addition, the loss of frataxin itself may initiate a coordinated resetting of transcriptional levels for many nuclear encoded proteins through a still unknown mechanism.

The expression of *PGC1alpha*, a master regulator of mitochondrial biogenesis, was unchanged, as was reported for the hearts of KIKO mice and for murine HL-1 cardiomyocytes in which frataxin was knocked down (Coppola et al., 2009), whereas Li et al. observed a decrease in an isogenic pair of iCMs (Li et al., 2019). The mRNA expression levels of master regulators of autophagy, such as *Bnip3* and *Becn1*, and of proteins involved in mitochondrial fusion (*Mfn1*, *Mfn2*, *Opa1*, *Drp1*), were also unchanged following frataxin knockdown. There are conflicting data about autophagy in different FRDA neuronal models, in which it was found to be either upregulated (Bolinches-Amoros et al., 2014; Simon et al., 2004) or unaffected (Palomo et al., 2011). In the hearts of both MCK conditional knockout mice (Huang et al., 2013) and conditional knockdown mice (Chandran et al., 2017), there is activation of autophagy. In these models, there is also activation of caspase 8 (Chandran et al., 2017) or caspase 12 (Huang et al., 2013). Autophagy may precede cardiomyocyte death during dilated cardiomyopathy (Shimomura et al., 2001). However, in both murine models, autophagy is detected weeks before death occurs, suggesting that these activation events can occur at low levels, at least for a time.

Variation in the mtDNA/nDNA ratio has been linked to cardiovascular disease (Castellani et al., 2020a) and reported in human FRDA heart tissue (Bradley et al., 2000). A slowly developing, extensive decrease in mtDNA/nDNA ratio has been reported in frataxin knockdown fibroblasts (Jasoliya et al., 2017), very similar to what we measured in NBT cells. However, the mtDNA/nDNA ratio in the cardiomyocytes decreased only ∼25%, suggesting a less-severe impairment, closer to what was reported for human FRDA heart tissue (Bradley et al., 2000). In the isogenic pair reported by Li et al. (2019), the mtDNA/nDNA ratio was unchanged.

In our bioinformatic analysis of transcriptomic data, the pathway most affected by frataxin knockdown was the type I interferon pathway, which was highly upregulated; a similar upregulation was reported in the inducible knockdown mouse model (Chandran et al., 2017) (in which there is no contribution of shRNA expression in the control mice), as well as in human FRDA fibroblasts, albeit with lower statistical significance (Napierala et al., 2017). Moreover, a large study of peripheral blood of FRDA patients identified “a transcriptional signature strongly enriched for an inflammatory innate immune response”, with gene sets that overlapped with those of the mouse model (Nachun et al., 2018). mtDNA has been linked to the activation of immune responses through multiple mechanisms (Riley and Tait, 2020), and different insults to mitochondrial stability can result in release of mtDNA into the cytosol (West et al., 2015).

Our data support the hypothesis that decreased frataxin activates a cell-intrinsic innate immune response. We observed mtDNA release into the cytosol following frataxin knockdown, as well as upregulation of IFNB1, mostly, although not entirely, through the activation of the cGAS-STING pathway. Increased cytosolic mtDNA was also observed in immortalized fibroblasts derived from FRDA patients. The increase in cytosolic mtDNA was less pronounced in the FRDA fibroblasts than in the transfected cells, suggesting that the presence of siRNAs may contribute to mitochondrial stress and mtDNA release, or that the sharp decline in frataxin level creates ‘fragile’ mitochondria, which are more easily disrupted during the mtDNA isolation process. Alternatively, chronically decreased frataxin levels may lead to a compensated state that includes some stabilization of mitochondria and resistance to mtDNA release, thereby keeping the interferon response in check. Regardless, our data show that decreased frataxin sensitizes mitochondria and makes them more prone to mtDNA release. Importantly, the increase in cytosolic mtDNA we observed was (1) in cells that are otherwise unstressed and not particularly reliant on mitochondria for their function, and (2) at a relatively low level, which is consistent with a chronic process and likely to be more relevant *in vivo*, as observed in other diseases (Amor et al., 2014).

A limitation of our model is that it is impossible to eliminate completely the contribution of transfected siRNAs to the phenotypes we observe. Events downstream of siRNA transfection and frataxin knockdown, such as upregulation of specific ISGs or activation of caspases, might be induced by a combination of multiple pathways. The analysis of the iCM transcriptomic data identified clusters of genes related to the cytosolic pattern-recognition receptor (PRR) activation, in particular RIG-1. Following frataxin knockdown in NBT cells and fibroblasts, we observed an upregulation of IFNλ, which is typically induced, together with *IFNB1*, by binding of RNA to RIG-1-like receptors (Brubaker et al., 2015). The transcriptomic data also showed an upregulation of caspase 1, an ‘inflammation’ caspase, which is typically activated by assembly of the inflammasome. Multiple factors could activate the inflammasome, including release of mtDNA (Pereira et al., 2019) and increased oxidative stress (Heid et al., 2013). Finally, although we saw a similar increase in cytosolic mtDNA in both NBT cells and fibroblasts following frataxin knockdown, downstream events might be more cell-type specific. Although fibroblasts can be useful to model FRDA, many questions about FRDA cellular abnormalities need to be explored further in iCMs derived from patient cells.

In conclusion, we developed a novel isogenic model of iCMs to study FRDA cardiomyopathy. Our data are consistent with previous results, confirm the suitability of the model, and reveal a novel pathway that may contribute to the pathophysiology of the disease and that suggests points of therapeutic intervention.

## MATERIALS AND METHODS

### Cells

Reprogramming of human, apparently healthy fibroblasts GM08399 (F, 19 years old; Coriell, Camden, NJ, USA), GM21808 (M, 1 day old; Coriell), the patient-derived FRDA line (F, 21 years old; GAA repeats 790/1120) and the zinc-finger-nuclease-corrected FRDA line were described in Li et al. (2015). All these lines were obtained from the Napierala laboratory at the University of Alabama at Birmingham (recently moved to UT Southwestern Medical Center). GM00942 cells (F, 5 years old; Coriell) and SV20 cells (M, 43 years old) were reprogrammed to iPSCs as described previously (Shanmughapriya et al., 2018; Yang et al., 2008) and were obtained from the iPSC Core at the University of Pennsylvania. Line SV20 (Penn123i-SV20) has been deposited at Wicell (https://www.wicell.org/home/stem-cells/catalog-of-stem-cell-lines/penn123i-sv20.cmsx?closable=true).

Differentiation into cardiomyocytes of all iPSC lines was performed at the iPSC Core Facility at the University of Pennsylvania as described previously (Laflamme et al., 2007; Palpant et al., 2017; Shiba et al., 2012). Differentiated cardiomyocytes were maintained in RPMI (#10-040-CM, Corning, Corning, NY, USA) plus B27 supplemented with insulin (#17504-044, Gibco, Waltham, MA, USA). Cells were tested for mycoplasma before differentiation.

Human, apparently healthy primary fibroblasts GM08400 were from Coriell and were grown in Dulbecco's modified Eagle medium (DMEM; #11885-076, Life Technology, Carlsbad, CA, USA) supplemented with 10% fetal bovine serum (FBS; #SH30910.03 Hyclone, GE Healthcare, Logan, UT, USA) and 1% penicillin/streptomycin (#15140 Life Technology). Human myoblast line NBT was described previously (Cudré-Mauroux et al., 2003) and was grown in Ham's F12 (#11765, Gibco) supplemented with 10% FBS (#SH30910.03 Hyclone, GE Healthcare), 1% penicillin/streptomycin (#15140, Life Technology), 100 µg/ml sodium pyruvate (#P4562), 1 mM creatine anhydrous (#C0780) and 50 µg/ml uridine (#U3003), all from Sigma-Aldrich (St Louis, MO, USA).

Immortalized lines are indicated with the name of the parental line and a ‘-T’ added to the end of the name. Control lines GM03348 (M, 10 years old) and GM03956 (F, 27 years old) were from Coriell. FRDA lines 4675 (M, 28 years old; GAA repeats 457/684), FRDA 6247 (F, 33 years old; GAA repeats 478/1017) were from the FRDA cell repository (University of Alabama at Birmingham). These cells were immortalized using the h-Tert immortalization kit with puromycin selection at 1 µg/ml as per the manufacturer's instructions (Alstem, Richmond, CA, USA), similarly to what we reported previously for cells 8399-T (F, 19 years old) and 3665-T (F, 13 years old; GAA repeats 790/1357), originally from Coriell (Cotticelli et al., 2020). A control/FRDA pair refers to cells that were immortalized at the same time, i.e. were infected, selected and grown for the same amount of time. Each pair was prepared separately.

### Frataxin knockdown

iPSC-derived cardiomyocytes at day 25 (±2 days) post differentiation were dissociated with trypsin 0.25%/EDTA (#25200, Gibco) and counted using the automated cell counter Countess© (Thermo Fisher Scientific, Philadelphia, PA, USA). At this time, an aliquot of cells was fixed and used to determine the percentage of cardiac troponin positivity. Cells were re-plated at the appropriate density in micro-wells previously coated with 0.1% gelatin (#ES-006-B, Millipore-Sigma, Burlington, MA. USA) in RPMI (#10-040-CM, Corning) containing 20% FBS (#SH30910.03 Hyclone, GE Healthcare) and 1 µM thiazovivin (#S1459, Selleckchem, Houston, TX, USA). The following day, medium was replaced with RPMI plus B27 supplemented with insulin, and the cells were allowed to recover for 2-3 days. Routinely, medium was changed every other day for the entire length of the experiment. GM08400 fibroblast were seeded at day 0 in 100 mm dishes at 200,000 cells/dish; NBT cells were seeded in 100 mm dishes at 80,000 cells/dish and transfected as reported below. Double-stranded siRNAs against frataxin mRNA (#SR301654-A, Origene, Rockville, MD, USA) or a control construct (sc 5′-rCAGUUUGCCCGGGAACCCACGGCGUGA-3′) were transfected using the Lipofectamine RNAi-Max reagent (#13778, Life Technology) at a final concentration of 10 nM as per the manufacturer's instructions. The other siRNAs against frataxin mRNA used were as follows: #SR301654-C, #SR320201-A (D), #SR320201-B (E), and #SR320201-C (F), all from Origene. The same transfection mix was used to transfect multiple types of dishes. We kept the notation sc1/siFxn1 or sc2/siFxn2 to indicate the number of transfections in the experiment shown.

In the experiments in which RU521 (#SML2347, Sigma-Aldrich) was used, drug was added at day 2 and day 6 at 2 µM final concentration. In the experiments with fibroblasts, drug was added 24 h before collecting the cells. The extent of frataxin knockdown was measured by ELISA (#176112, Abcam, Waltham, MA, USA) as per the manufacturer's instructions at 48 h post-transfection. A scheme of the experiment is shown in Fig. S1C. In the experiments in which cells were only transfected once, the first transfection was performed at day 5. The majority of experiments were performed with cells collected around day 31-34 post-differentiation, and no experiments were run after day 40 post-differentiation.

### Flow cytometry

iCMs were fixed in 1% paraformaldehyde (PFA; #19943, Affymetrix, Cleveland, OH, USA), permeabilized with Saponin (#557885, BD Bioscience, San Jose, CA, USA) and stained for cardiac troponin using FITC-conjugated anti-cardiac troponin antibody (Arshi et al., 2013) at dilution 1:8 (#130-106-687, Clone REA400, Militeny Biotech, Auburn, CA, USA) as per the manufacturer's instructions. The NBT cells transfected twice with sc2 or siFxn2 were fixed in 4% PFA , permeabilized and stained with a mouse monoclonal antibody anti-dsRNA clone rJ2 (#MABE1134, Millipore-Sigma, St Louis, MO, USA), used at 1:200 dilution as described in Dhir et al. (2018). Secondary antibody was goat anti-mouse Alexa Fluor 488 used at 1:1000 dilution. (#2015565, Thermo Fisher Scientific). Data were acquired with a LSR Fortessa (BD Bioscience) and analyzed using FlowJo v.10.7.1 (BD Bioscience).

### Transcriptome analysis

iPSC-derived cardiomyocytes were transfected twice with siFXN or the control siRNA as described above. RNA was extracted at day 7 using an mRNAeasy kit (#74106, Qiagen, Germany). RNA quality control, library construction and sequencing were performed at the Next Generation Sequencing Core at the University of Pennsylvania. Raw (fastq) data were imported into Salmon (https://combine-lab.github.io/salmon/; Patro et al., 2017) to count hits against the transcriptome defined in Gencode v35 (https://www.gencodegenes.org). Further analysis was done in R (https://cran.r-project.org/). Hits for each sample were annotated and summarized to the gene level with tximeta (https://bioconductor.org/packages/release/bioc/html/tximeta.html; Love et al., 2020) and further annotated with bioMARt (https://bioconductor.org/packages/release/bioc/html/biomaRt.html; Durinck et al., 2005, 2009). Gene level data were normalized and analyzed statistically with DESeq2 (https://bioconductor.org/packages/release/bioc/html/DESeq2.html; Love et al., 2014). PCA was plotted using pcaExplorer (https://bioconductor.org/packages/release/bioc/html/pcaExplorer.html; Marini and Binder, 2019). Genes were considered significant with a 1.5-fold change up or down, and a false-discovery rate (*P*-adj)≤0.05. The list of significant genes was further analyzed for enrichment of pathways and gene sets using IPA (https://digitalinsights.qiagen.com/IPA; Krämer et al., 2014). Data and IPA analysis were performed by the Molecular Profiling Facility at the University of Pennsylvania. Original data files and details about the procedure used are available in the ArrayExpress database (www.ebi.ac.uk/arrayexpress) under accession number E-MTAB-11296.

### Seahorse analysis

OCRs (pmol/min) were determined using the Seahorse XF96 Extracellular Flux Analyzer (Agilent, Santa Clara, CA, USA) following the manufacturer's protocol. iCMs were seeded at a density of 5×10^4^ cells/well (cell-seeding density was optimized in preliminary experiments) in an XF96-well microplate previously coated with 0.1% gelatin (#ES-006-B, Millipore-Sigma). Medium was changed every other day starting from day 1. Cells were transfected with siFXN or a control construct at day 5 in a 100 µl final volume and assayed at day 7.

NBT cells were transfected as described above. Cells transfected with sc1 or siFxn1 were then seeded in the Seahorse plate at 50,000 cells/well and the assay was run the following day. Cell viability was assessed post-assay and the results were used to normalize the OCR measurements. The day of the assay, medium was replaced with XF assay medium supplemented with 1 mM sodium pyruvate (#P4562, Sigma-Aldrich), 2 mM glutamine (#25030, Gibco), 5 mM glucose (#9432, Sigma-Aldrich) and 10% FBS (#SH30910.03 Hyclone, GE Healthcare) at pH 7.4. Cells were equilibrated in a non-CO_2_ incubator at 37°C for 1 h. Cells were then placed in the instrument, and oxygen consumption was recorded for almost 90 min. OCR was measured simultaneously in all wells three times at each step, and a minimum of eight replicates were utilized per condition in any given experiment. Basal OCR was measured, followed by OCRs after sequential treatment with oligomycin A (1 μM), carbonylcyanide-*p*-trifluoromethoxyphenylhydrazone (FCCP; 0.25 μM) and rotenone/antimycin (1 μM). (Optimal reagent concentrations were determined in preliminary experiments.) All compounds and materials were obtained from Agilent.

### Mitogreen staining

Three-hundred-thousand iCMs were seeded on a 29 mm glass-bottom (10 mm) dish (Cellvis, #D29-10-1.5-N, Sunnyvale, CA, USA). The following day, live cells were incubated with 0.5 µg/ml Mitogreen (#M7514, Thermo Fisher Scientific, Waltham, MA, USA) for mitochondria in medium at 37°C for 30 min. For NBT cells, 50,000 cells were seeded in an eight-chamber slide (#80826, Ibidi GmbH, Gräfelfing, Germany). The following day, live cells were incubated with 0.5 µg/ml Mitogreen (#M7514, Thermo Fisher Scientific) for mitochondria in medium at 37°C for 30 min together with Hoechst (#33342, Thermo Fisher Scientific) at 1 µg/ml final concentration. Images were taken by confocal microscopy (Zeiss LSM710, software Zen2.1) using excitation of 490 nm and emission of 516 nm for Mitogreen and excitation of 405 nm for Hoechst.

### Troponin/propidium iodide staining

Forty-thousand iCMs were seeded in one well of an eight-chamber slide (#155409 Lab-TekII, Thermo Fisher Scientific). Cells were fixed with 4% PFA in PBS, and blocked with 5% FBS, 0.3% Triton-X (#BP151-100, Fisher Scientific, Waltham, MA, USA) and 1% bovine serum albumin (#SLCB8319, Sigma-Aldrich) in PBS (#21030, Corning) overnight. Cells were stained with mouse anti-cardiac troponin (Gladka et al., 2021) used at 1:400 dilution (ab8295, Abcam, Waltham, MA, USA). Secondary antibody was goat anti-mouse Alexa Fluor 488 used at 1:1000 dilution (#A11001, Invitrogen). Cells were washed twice with PBS, and one drop of propidium iodide (#R37108, Molecular Probes, Invitrogen) was added. The slide was treated with Anti-Fade Fluorescence Mounting Medium (ab104135, Abcam) and imaged with a Zeiss fluorescent microscope (Zeiss AxioVision 4.8 software).

### JC-1 assay

Three-hundred-thousand iCMs were seeded on a 29 mm glass-bottom (10 mm) dish (Cellvis, #D29-10-1.5-N, Sunnyvale, CA, USA) and allowed to recover for 2 days. The medium was removed, 1 ml JC-1 (#T3168, Thermo Fisher Scientific) solution (1 µg/ml final concentration) was added, and cells were returned to the incubator for 25 min. Cells were then washed gently with medium once and visualized. Images were taken by confocal microscopy (Zeiss LSM710, Zen2.1 software) using 488 nm excitation and 535 nm (monomers) and 610 nm emission (aggregates). The ratio of aggregates/monomers was quantified using ImageJ software (National Institutes of Health, Bethesda, MD, USA; http://imagej.nih.gov/ij). Outlines were drawn around the cells, and area, integrated density and mean fluorescence (including background areas) were measured. To calculate the CTCF, we used the following formula:
(1)

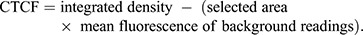


Averages of five areas were used for each image.

### Mitochondria copy number

DNA from iCMs was extracted from cell pellets collected at day 7, following two transfections with siRNA. Cell lysis (100 mM Tris-HCl pH 8.5, 0.5 mM EDTA, 10% SDS, 5 M NaCl) was followed by overnight digestion with Proteinase K (20 mg/ml) at 55°C. DNA was precipitated by adding one volume of isopropanol and isolated by centrifugation at 20,000 ***g*** for 15 min at 4°C. DNA was washed with 70% ethanol, resuspended in 10 mM Tris-HCl pH 7.5, and quantified by optical density using NanoDrop One (Thermo Fisher Scientific). For each PCR reaction, 50 ng DNA was used. Human B2 microglobulin (B2M) was used to normalize the results.

### Cytosolic mtDNA extraction

Cytosolic mtDNA was extracted as described previously (Bronner and O'Riordan, 2016). Briefly, cells contained in one 100 mm dish were scraped with 300 µl of 1% NP-40 and centrifuged at 15,000 ***g*** at 4°C for 15 min. Cytosolic DNA was isolated using a DNeasy Blood and Tissue Isolation kit (#69504, Qiagen, Germany) and quantified by optical density using NanoDrop One. At least 50 ng DNA was used for each PCR reaction. Human 18S was used to normalize the results.

#### Real-time PCR

Total RNA was extracted using the RNeasy Mini kit (#74106 Qiagen, Germany) and quantified by optical density. One microgram of total RNA was retro-transcribed using the QuantiTect Reverse Transcription kit (#205313, Qiagen, Germany). Levels of DNA or expressed mRNA were assessed by Real-Time PCR using a TaqMan assay. Primers and reagents were from Applied Biosystems (Thermo Fisher Scientific). Samples were run in triplicate and analyzed using the Delta C(T) method (2-ΔΔCt). Unless otherwise noted, human *TBP* was used to normalize the results. The expression level of controls was assumed to be 1, and the changes were calculated accordingly. Primers used are listed in Table S1.

### Data and statistical analysis

Experiments performed with iCMs were optimized using mostly lines SV20 and 21880. Once the conditions were determined and the results were reproducible, the experiments were repeated in the other lines (Fig. 1 and Fig. S4).

When analyzing four lines, weighted averages of four biological replicates (µ) and SD (σ) were calculated using the following formulae:
(2)

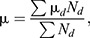

(3)

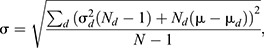
where µ*_d_* is the average for each line, *N_d_* is the number of technical replicates for one line, and *N* is the total number of replicates 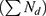. *P*-values (unpaired, two-tailed) were calculated using the weighted averages, s.d. and *N* by Prism (v 5.03; GraphPad). Statistical analyses were performed using Prism (v 9.3.1; GraphPad).

## Supplementary Material

10.1242/dmm.049497_sup1 Supplementary information
