# Supplementary material for: Acute frataxin knockdown in induced pluripotent stem cell-derived cardiomyocytes activates a type I interferon response
Source: Dis Model Mech. 2022 Oct 26;16(5):dmm049497. doi: 10.1242/dmm.049497 (PMC9637271; doi:10.1242/dmm.049497)
Supplement: Supplementary information [file dmm-16-049497-s1.pdf]

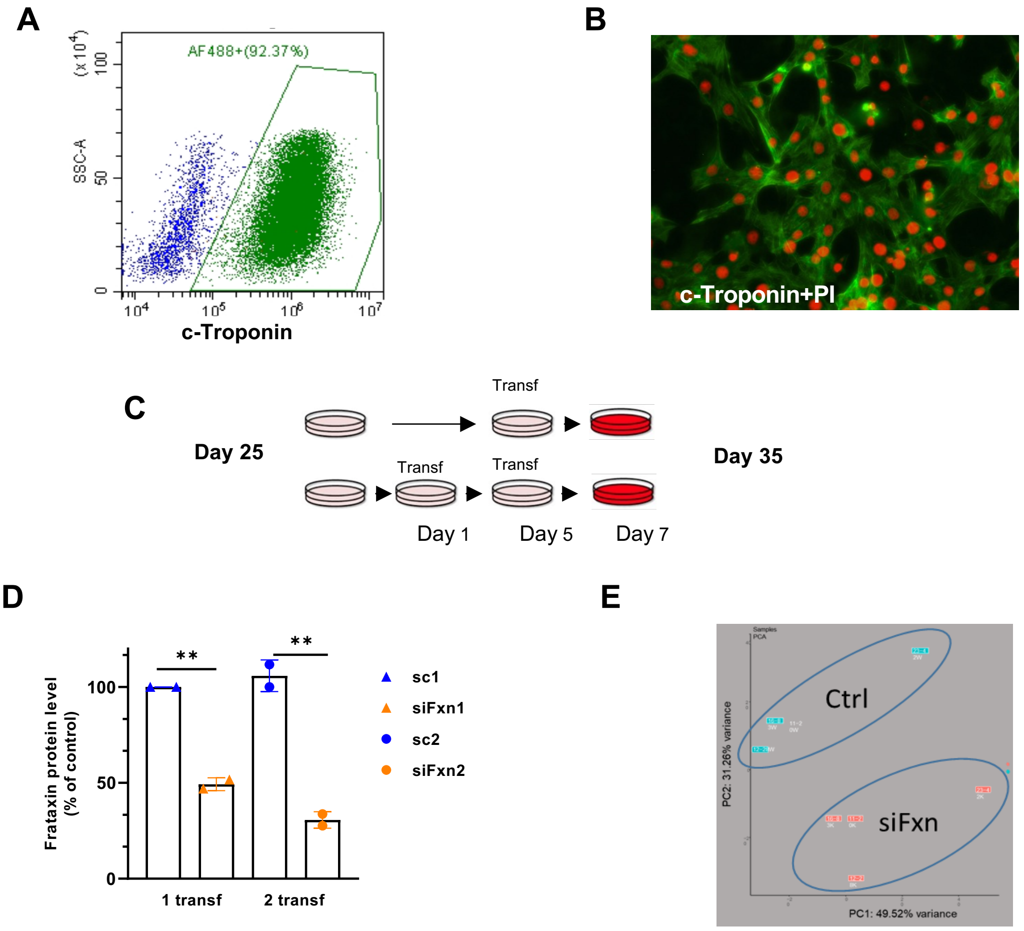

Fig. S1. Frataxin knockdown in human iCMs

(A) iPSC differentiation into cardiomyocytes. Representative experiment showing % of iCMs that are cardiac troponin positive at day 25.

(B) Cardiac troponin (green) and nuclei (red) IHC staining (20x) of iCMs at day 25 post differentiation.

(C) Flow chart of transfection experiments. Differentiated iCMs seeded in their final vessel were either transfected once, on day 5, or transfected twice, on day 1 and day 5. All cells were harvested at day 7.

(D) Representative experiment showing frataxin protein level measured by ELISA 48 hours after one transfection or two transfections of iCMs. The data show the mean ± 1 SDs of two independent experiments, each one in triplicate. *** indicates $p < 0.005$, calculated by unpaired, two-tailed, Student's t test.

(E) Transcriptome principal component analysis of the four biological replicates after two transfections.

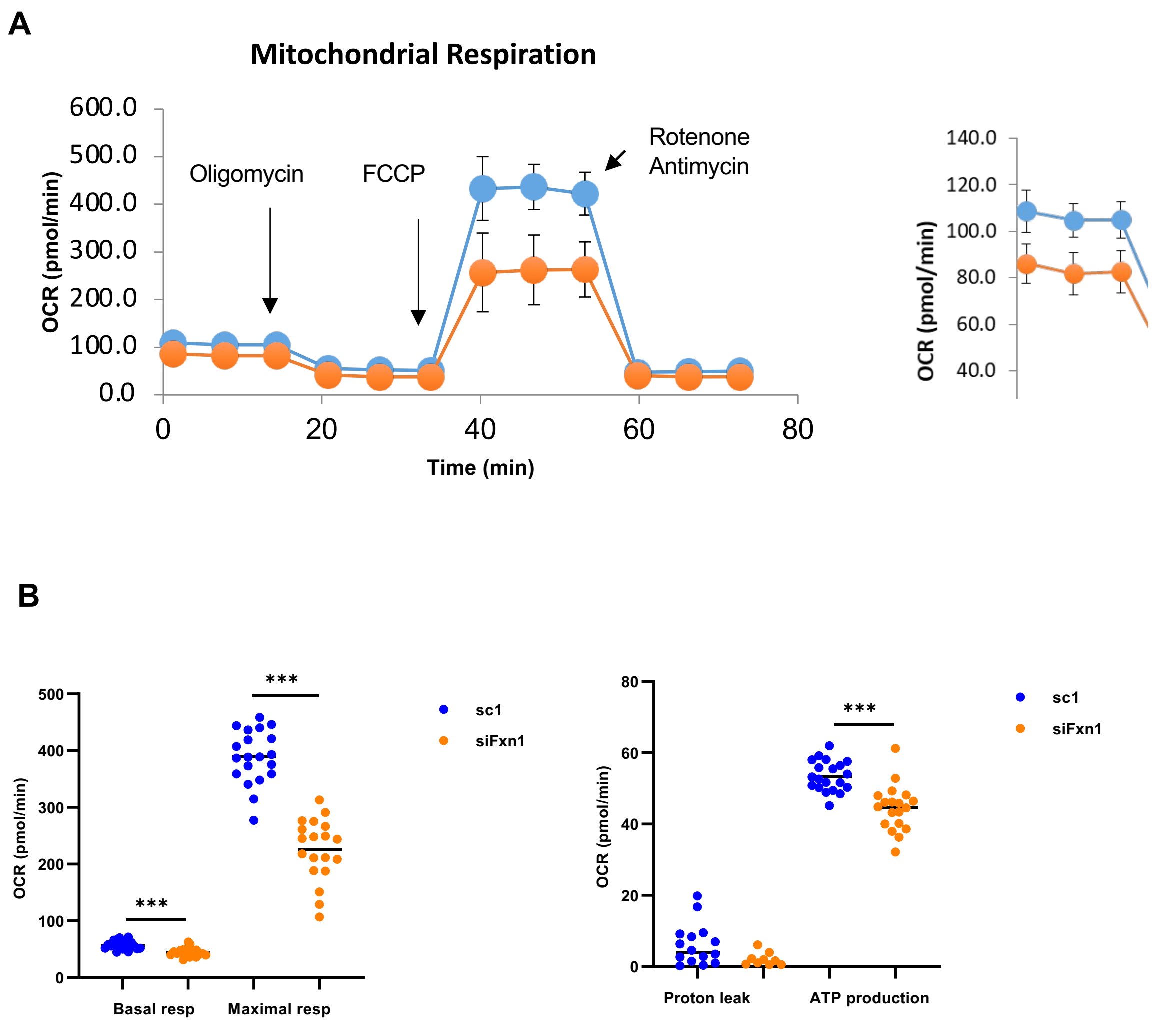

Disease Models & Mechanisms • Supplementary information

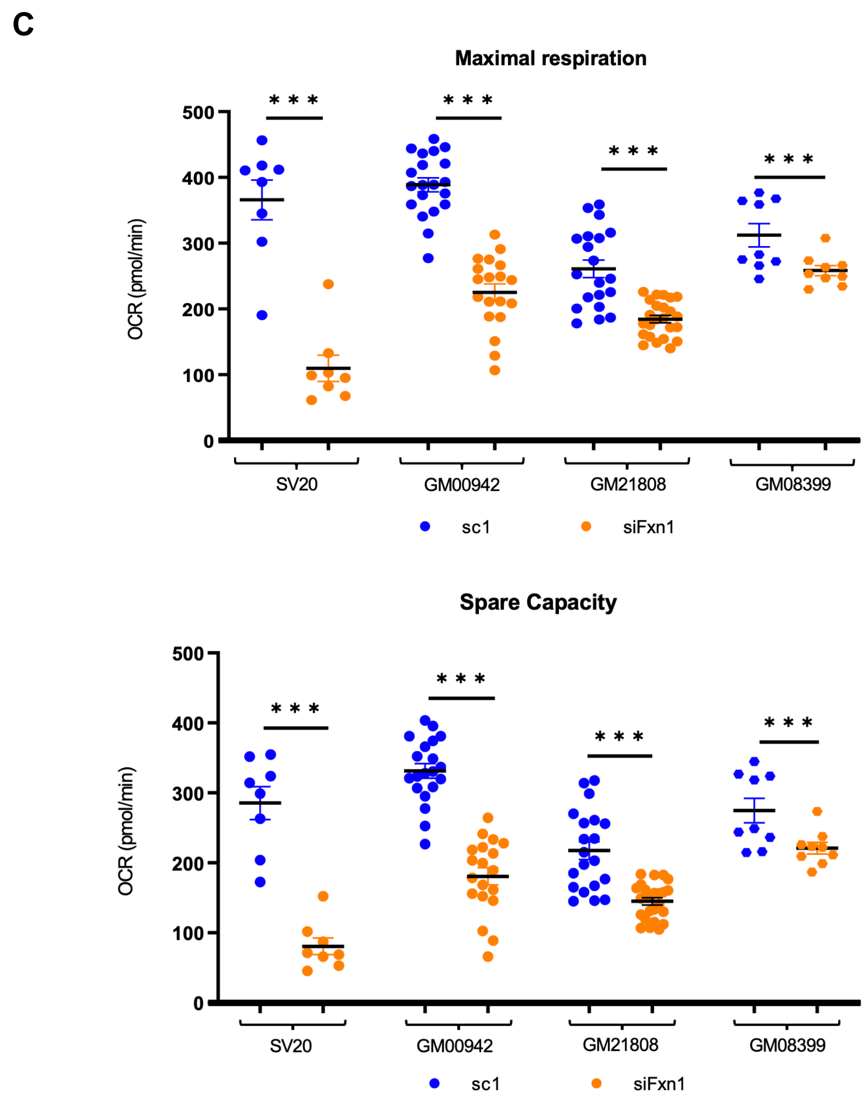

Fig. S2. Frataxin knockdown impairs mitochondrial respiration in iCMs.

(A) Seahorse profile of GM00942 iCMs independently transfected once with siFxn (orange, n = 19) or sc control (blue, n = 20). SD bars at basal level, after oligomycin injection and after rotenone/ antimycin injection, are only visible on a blown-up scale (right side).

(B) Differences in key parameters (sc in blue; siFxn in orange). *** = p < 0.001 by unpaired, two-tailed Student's t test. Data are represented as means ± 1 SD.

(C) Maximal respiration (top) and spare capacity (bottom) are lower in cells transfected once with siFxn (siFxn2; orange) vs. control (sc1; blue) in each iCM line. Data are represented as means ± 1 SD. *** = p < 0.005 calculated by unpaired, two-tailed Student's t-test. Numbers of independently transfected replicates for sc1: SV20, n = 8; GM942, n = 20; 21808, n = 20; GM8399 n = 9; and for siFxn1: SV20, n = 8; GM942, n = 20; 21808, n = 20; GM8399, n = 19.

Disease Models & Mechanisms • Supplementary information

A

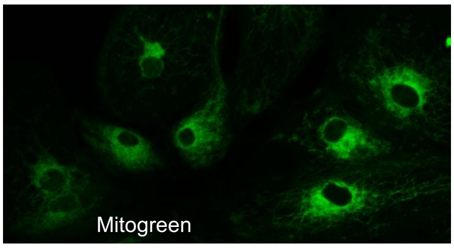

B

	mt DNA /n DNA (% of control)		
	mt Nd1	mt Nd5	mt CO1
SV20 +siFxn2	11.6±1.0	30.5±6.7	67.3±9.1
GM00942+siFxn2	76.6±8.7	81.8±7.1	85.3±4.2
GM28808+siFxn2	71.6±11.8	79.7±7.2	72.1±10.2
GM08399+siFxn2	55.4±0.6	64.2±14.7	57.2±0.8

C

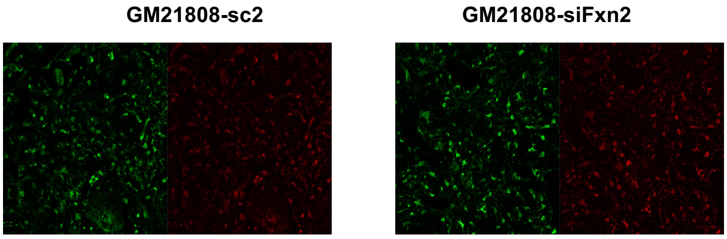

D

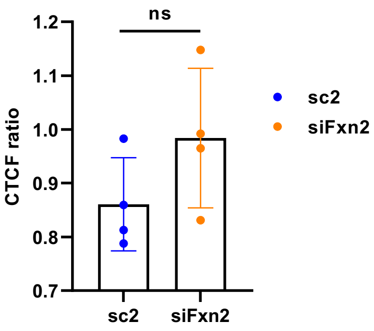

Fig. S3. Mitochondria morphology and copy number in iCMs

(A) Representative Mitogreen staining (20x) in iCMs.

(B) mtDNA/nDNA ratio decreases in all four iCM lines following frataxin knock down. Results are expressed as % of control ± 1SD. The difference between sc2 and siFxn2 are significant: p value < 0.0001 by two-way ANOVA.

(C) Mitochondrial Membrane potential in iCMs (20x) transfected twice with siFxn (right) or a control siRNA (left). Cells were loaded with the fluorescent dye JC1, which is imported into mitochondria where it aggregates. In each picture CTCF (corrected total cell fluorescence) in each channel was calculated as reported in the Methods section. The ratio of fluorescence signals (red[mito]/green[cytosolic]) was calculated for at least five different areas.

(D) Weighted average of the CTCF ratio ± 1SD of 4 biological replicates for cells transfected with sc2 (blue, n=22), and for cells transfected with siFxn2 (blue, n=22).

Disease Models & Mechanisms • Supplementary information

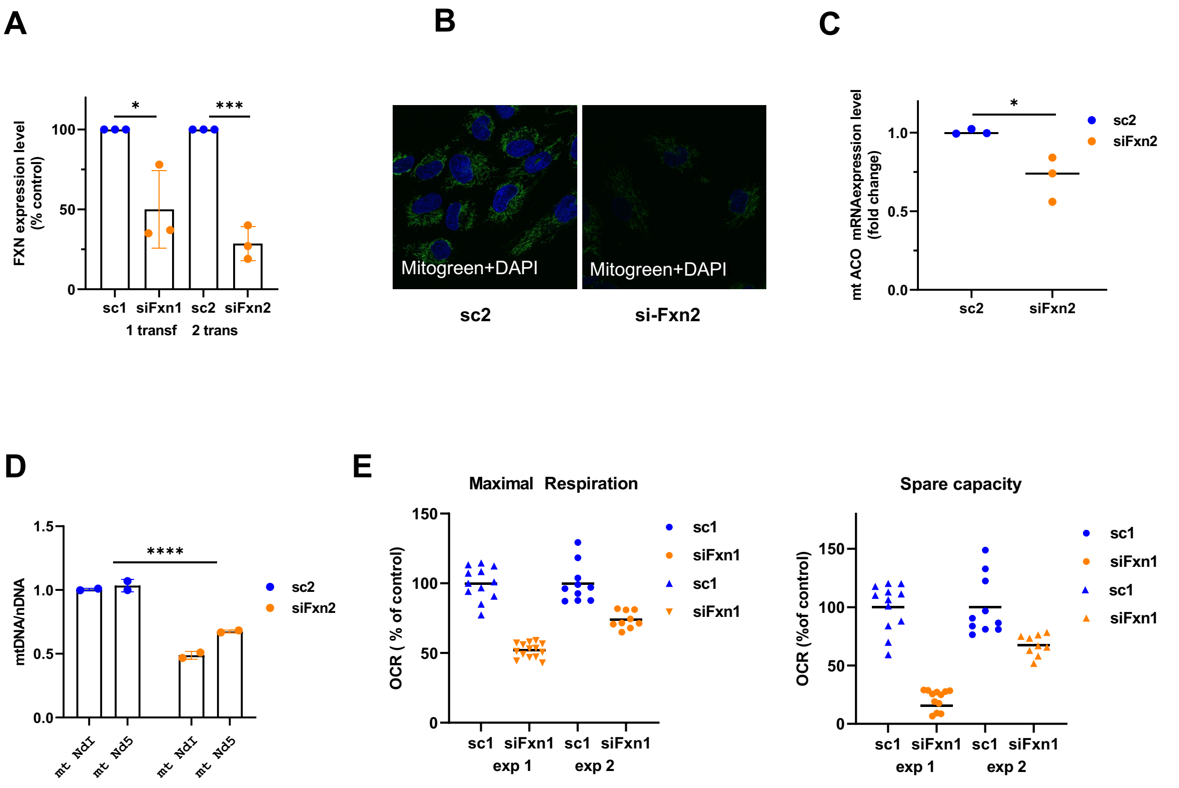

Fig. S4. Frataxin knockdown in NBT cells

Data show the mean ± 1SD of three (A,C) or two independent experiments (D), each one in triplicate.

(A) Frataxin protein level measured 48 hours after one transfection or two transfections with siFxn (orange) or sc control (blue). * = $p < 0.05$; *** = $p < 0.005$, calculated by unpaired, two-tailed, Student's t test.

(B) Representative Mitogreen staining (63x) of NBT cells transfected with siFxn2 (right) or sc2 (left).

(C) Mitochondrial aconitase expression level in NBT cells transfected twice with sc2 (blue) or siFxn2 (orange). * indicates $p < 0.05$ by unpaired, two-tailed, Student's t test.

(D) mtDNA/nDNA (*B2M*) ratio decreased in NBT cells transfected twice with siFxn2 (orange) vs control sc2 (blue). *** indicates $p < 0.0001$ by two-way ANOVA.

(E) Maximal respiration (left) and Spare respiratory capacity (right) measured in the Seahorse assay in NBT cells transfected once with siFxn1 (orange) or sc1 (blue). Number of replicates for experiment 1: sc1 n=10, siFxn1 n= 9; for experiment 2: sc1 n=12, siFxn1 n=13. Differences between sc1 and siFxn1 for both measurements are significant ($p < 0.001$ by two-way ANOVA).

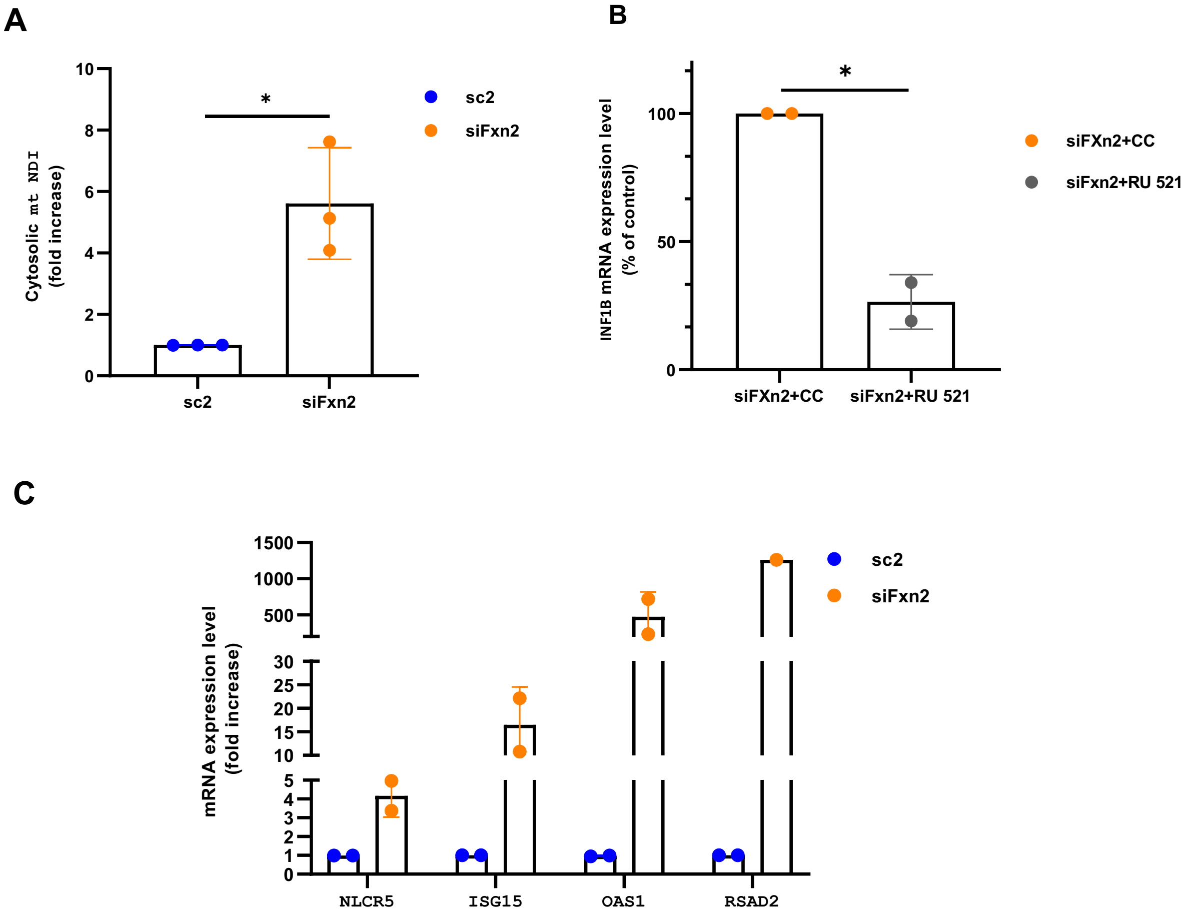

Fig. S5. Type I interferon response follows frataxin knockdown in primary human fibroblasts

Data show mean ±1 SD of triplicate (A) or duplicate (B,C) experiments, each one at least in duplicate.

(A) Cytosolic concentration of mitochondrial DNA (mtDNA) increases with frataxin knockdown. Cytosolic mtDNA was extracted from 8400 cells transfected twice with siFxn2 (orange) or control sc2 (blue). *mtNd1* was amplified and its concentrations was normalized using 18S DNA. * indicates $p < 0.05$ by unpaired, two-tailed Student's t test.

(B) *INF1B* expression can be detected in 8400 cells transfected twice with siFxn (siFxn2, Ct values <32) but is barely expressed in cells transfected with control (sc2, Ct values >35). Treatment with the cGAS inhibitor RU521 at 2 µM after each transfection significantly decreases IFN1B expression. * indicates $p < 0.05$ by unpaired, two-tailed, Student's t test.

(C) Upregulation of ISGs *NLRC5*, *ISG15, OAS1* and *RSAD2* in 8400 cells transfected twice with siFxn (siFxn2, orange) or control (sc2, blue). Differences between sc2 and siFxn2 are significant: p = 0.0078 by two-way ANOVA.

Disease Models & Mechanisms • Supplementary information

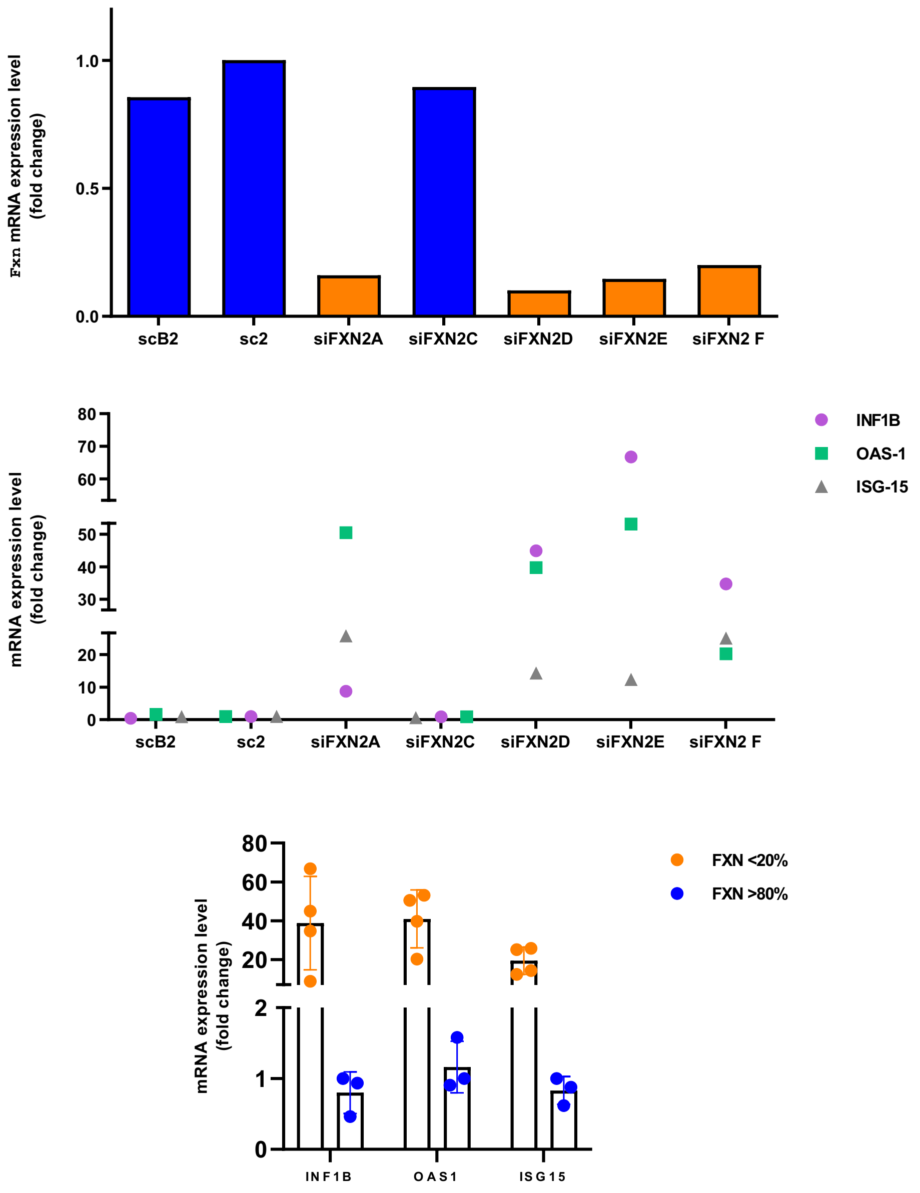

Fig. S6. Type I interferon response follows frataxin knockdown.

NBT cells were transfected twice with two control siRNAs and five siRNAs targeting *FXN* mRNA. In the cells where *FXN* mRNA was knocked down (top panel) there was upregulation of *INF1B*, *OAS-1,* and *ISG15* mRNAs (middle panel). The differences in expression levels of *IFN1B*, *OAS-1*, and *ISG15* in transfected cells in which *FXN* mRNA expression levels were >20% of control were significantly higher that in cells in which frataxin was >80% of control (bottom panel) with $p < 0.005$ by two-way ANOVA.

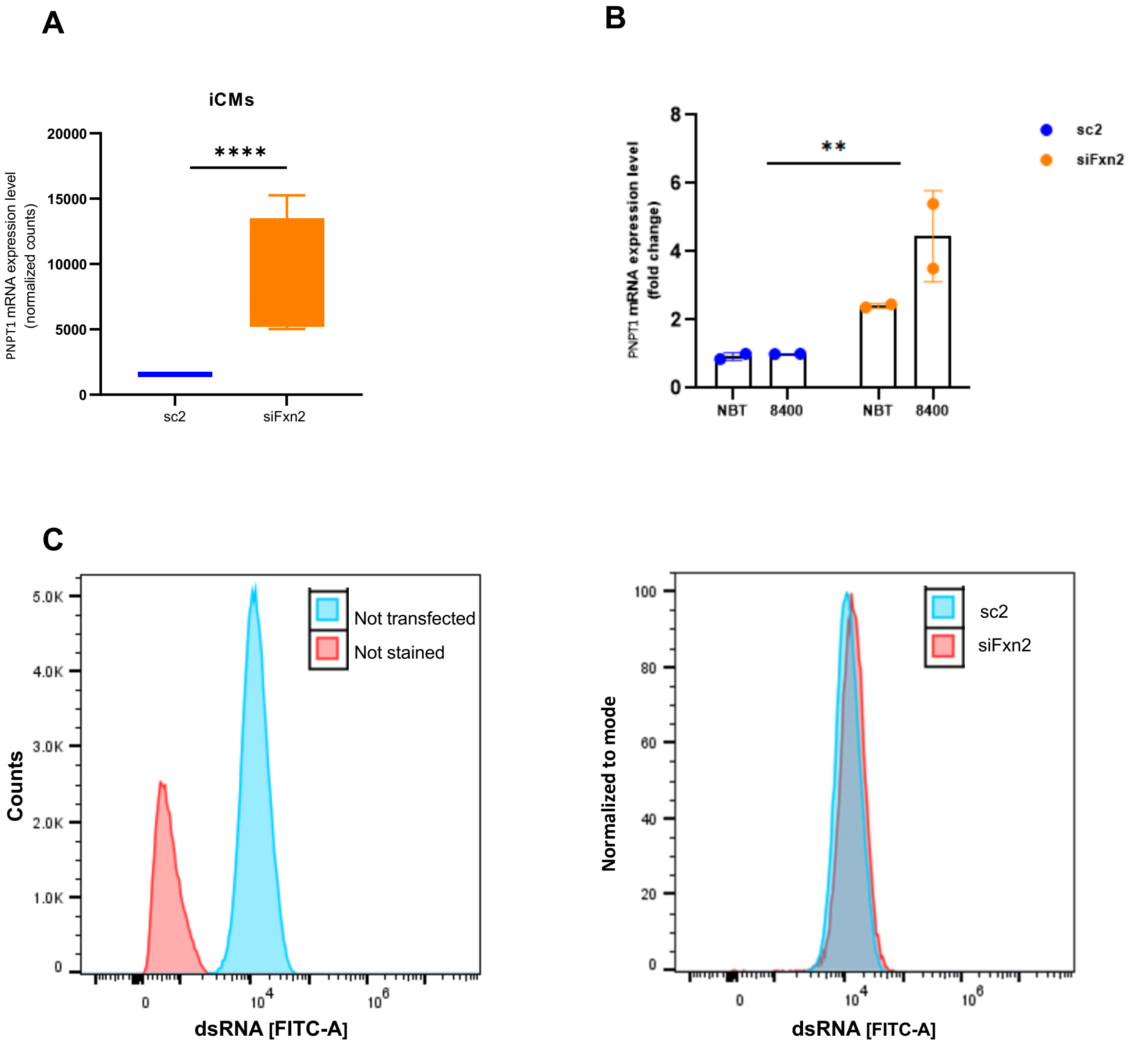

Fig. S7. Detection of dsRNA does not change following Frataxin knockdown

(A) Box and Whisker plots showing normalized counts for *PNPT1* in iCMs transfected with control sc2 (blue) or siFx2 (orange). Number of biological replicates = 4; **** indicates p-adj < 0.0001.

(B) *PNPT1* expression levels decreased in human fibroblasts 8400 (left) and NBT cells (right) transfected twice with siFxn (siFxn2 orange) or control (sc2 blue). The results shown are the averages and the SDs of two independent experiments, each one in triplicate. ** indicates p < 0.01 by two-way ANOVA.

(C) dsRNA can be detected in NBT cells not transfected (left). Transfection with either sc2 (blue) or siFxn2 (red) did not increase median fluorescence.

Table S1. Primers used (#4331182, Thermo Fisher, Waltham, MA)

Hs01077958-s1	IFNB1
Hs00601677_g1	IFNL1
Hs01072123_m1	NLRC5
Hs00973635_m1	OAS1
Hs003698123-m1	RSAD2
Hs01921425_s1	ISG15
Hs1105971_m1	PNPT1
Hs02596873_s1	MT-ND1
Hs02596878-g1	MT-ND5
Hs02596864_g1	MT-CO1
Hs00426616_g1	Aco2
Hs00175940_m1	FXN
Hs00427620_m1	TBP
Hs99999901	18S
Hs06637353_s1	B2M
